# A comparative study of extracellular vesicle-associated and cell-free DNA and RNA for HPV detection in oropharyngeal squamous cell carcinoma

**DOI:** 10.1038/s41598-020-63180-8

**Published:** 2020-04-08

**Authors:** Bella Nguyen, Katie Meehan, Michelle R. Pereira, Bob Mirzai, Si Hong Lim, Connull Leslie, Michael Clark, Chady Sader, Peter Friedland, Andrew Lindsay, Colin Tang, Michael Millward, Elin S. Gray, Annette M. Lim

**Affiliations:** 1 Department of Medical Oncology, Sir Charles Gairdner Hospital, Perth, Western Australia Australia; 2 School of Biomedical Sciences, University of Western Australia, Perth, Western Australia Australia; 3 Chinese University of Hong Kong, Shatin, Hong Kong; 4 School of Medical and Health Sciences, Edith Cowan University, Joondalup, Western Australia Australia; 5 Genomics Western Australia, Telethon Kids Institute, Western Australia Perth, Australia; 6 School of Pathology and Laboratory Medicine, The University of Western Australia, Perth, Western Australia Australia; 7 Department of Anatomical Pathology, PathWest, QEII Medical Centre, Perth, Western Australia Australia; 8 Department of Otolaryngology, Head and Neck Surgery, Sir Charles Gairdner Hospital, Perth, Western Australia Australia; 9 Department of Otolaryngology, Head and Neck Surgery, St John of God Murdoch Hospital, Perth, Western Australia Australia; 10 Faculty of Medical and Health Sciences, University of Western Australia, Perth, Western Australia Australia; 11 School of Medicine, University of Notre Dame, Fremantle, Western Australia Australia; 12 Department of Otolaryngology, Head and Neck Surgery, Hollywood Private Hospital, Perth, Western Australia Australia; 13 Department of Radiation Oncology, Sir Charles Gairdner Hospital, Perth, Western Australia Australia; 14 School of Medicine, The University of Western Australia, Perth, Western Australia Australia; 15 Department of Medical Oncology, Peter MacCallum Cancer Centre, Melbourne, Victoria Australia; 16 Sir Peter MacCallum Department of Oncology, The University of Melbourne, Melbourne, Victoria Australia

**Keywords:** Head and neck cancer, Tumour biomarkers

## Abstract

Purpose: This study compares the detection sensitivity of two separate liquid biopsy sources, cell-free (cf) DNA/RNA and extracellular vesicle (EV)-associated DNA/RNA (EV-DNA/RNA), to identify circulating Human Papilloma Virus (HPV) DNA/RNA in plasma obtained from patients with oropharyngeal squamous cell carcinoma (OPCSCC). We also report on the longitudinal changes observed in HPV-DNA levels in response to treatment. Experimental **design:** A prospective study was conducted that included 22 patients with locally advanced disease and six patients with metastatic OPCSCC. Twenty-three patients had HPV-related OPCSCC defined by p16 immunohistochemistry. Levels of circulating HPV-DNA and HPV-RNA from plasma-derived cf-DNA/RNA and EV-DNA/RNA were quantified using digital droplet PCR. Results: Circulating HPV-DNA was detected with higher sensitivity in cf-DNA compared to EV-DNA at 91% vs. 42% (*p* = <0.001). Similarly, circulating tumoral HPV-RNA was detected at a higher sensitivity in cf-RNA compared to EV-RNA, at 83% vs. 50% (*p* = 0.0019). In the locally advanced cohort, 100% (n = 16) of HPV-OPCSCC patients demonstrated a reduction in circulating HPV-DNA levels in cf-DNA following curative treatment, with 81% of patients demonstrating complete clearance to undetectable levels. However, in metastatic HPV-OPCSCC patients (n = 4), HPV-DNA levels did not correlate with treatment response. Conclusion: Our study demonstrates that although HPV-DNA/RNA can be detected in EV associated DNA/RNA, cf-DNA/RNA is the more sensitive liquid biopsy medium. As circulating HPV-DNA levels were found to only correlate with treatment response in the locally advanced but not metastatic setting in our small cohort of patients, the use of HPV-DNA as a dynamic biomarker to monitor treatment response requires further evaluation.

## Introduction

The Human Papilloma Virus (HPV) is an epitheliotropic virus that requires the environment of differentiating squamous epithelium for their cell cycle and is known to be directly implicated in the oncogenesis of multiple cancers including squamous cell carcinomas of the oropharynx^1^. HPV-driven oropharyngeal squamous cell carcinomas (HPV-OPCSCC) have been recognized to be a clinicopathologically distinct disease compared to non-HPV driven OPCSCC, as reflected by the different associated risk factors, pathological features and associated improved prognosis^2,3^. In the United States, over the past two decades, the incidence of HPV-OPCSCC has risen from 16.3% to as high as 71.7% of all OPCSCC cases^2,4^.

The rising incidence and clinical implications of HPV-OPCSCC demand improvement in the laboratory technologies available to accurately and efficiently identify tumoral HPV elements. HPV-OPCSCC is most commonly defined by the presence of overexpression of the cellular protein p16 detected using immunohistochemistry (IHC), which has been confirmed to be a robust surrogate marker of HPV-transformed OPCSCC cells^3^. Although sensitive, pragmatic and the current clinical standard of care, p16 overexpression can occur due to other somatic mutations and consensus guidelines refer to detection of HPV E6/E7 mRNA by *in-situ* hybridization (ISH) as a gold standard, as this detects the presence of transcriptionally active HPV elements^5^. However, the use of HPV-RNA ISH is associated with a number of challenges, including difficulties with adequate tissue preservation, higher costs of the test and the significant labour involved^6^. All current diagnostic strategies to confirm the presence of HPV-related disease remain reliant on the ability to obtain solid tumoral tissue from invasive biopsies.

Liquid biopsies offer a non-invasive approach to detect tumoral molecular elements, which can be performed serially with ease to monitor clinical progress. Cell-free (cf-DNA) is one of the fastest-growing liquid biopsy biomarkers that has been extensively validated and is currently used routinely in the clinical setting to test for specific tumoral mutations in oncogene driven cancers^7–11^. A number of studies have also demonstrated the emerging role of extracellular vesicles (EV) as another complementary liquid biopsy source^12–16^. EV are bioactive vesicles secreted by all cell types, including vesicles of various sizes and biogenetic origin (e.g. exosomes, microvesicles, oncosomes and microparticles) and are implicated in carcinogenesis and in the facilitation of metastatic potential^12^. EV have also been found to contain molecular content reflective of the characteristics of the tumor and thus, EV and their contents have the potential to represent a novel liquid biopsy biomarker^12–16^. As EV are hypothesized to contain more intact molecular contents, EV-based liquid biopsy approaches provide an alternative to cell-free based approaches which rely on the detection of degraded, apoptotic cellular material^17^.

This study aimed to compare the sensitivity of EV-associated DNA/RNA (EV-DNA/RNA) and cf-DNA/RNA in detecting tumoral HPV-DNA/RNA in plasma from OPCSCC patients compared to the current clinical standard of care being p16 IHC testing on slides from formalin-fixed paraffin-embedded tumoral (FFPE) biopsy samples. We also investigated the longitudinal changes of circulating tumoral HPV-DNA in cf-DNA and EV-DNA in HPV-OPCSCC patients undergoing treatment for locally advanced and metastatic disease.

## Materials and Methods

### Participants, ethics and consent

Twenty-two patients with locally advanced disease and six patients with metastatic (total n = 28) OPCSCC were recruited prospectively for the study between April 2016 and May 2018. HPV status was determined using p16 immunohistochemistry reported by accredited clinical diagnostic laboratories, which reported positive expression as diffuse staining with strong intensity. Two samples that were originally reported as p16 negative in the context of insufficient and necrotic tissue, had repeat p16 IHC testing performed at an independent reference laboratory service given liquid biopsy testing demonstrated the presence of HPV and were subsequently found to be positive. For all patients, blood samples were taken at baseline prior to treatment, and subsequently at approximately six weekly intervals during treatment. Longitudinal analyses for this study were performed on two serial samples per patient. For patients in the curative setting, samples were analyzed at baseline and then at the 6–12 week post-treatment time period around the first disease response assessment. For patients in the metastatic setting, samples were analyzed at baseline and at the third serial collection, ranging between 8–22 weeks from baseline collection. Tumor responses in metastatic patients were assessed radiologically at 8–12 weeks intervals using whole-body Computed Tomography (CT) scans. CT scans were assessed using the Response Evaluation Criteria in Solid Tumors (RECIST) 1.1 criteria and were classified as having a complete response (CR), partial response (PR), stable disease (SD) or progressive disease (PD)^18^.

The study received approval from the Sir Charles Gairdner Group Human Research Ethics Committee (HREC 2015-062) and Hollywood Hospital Ethics Committee (HPH498) and was conducted according to the principles set out by the National Statement on Ethical Conduct in Human Research and Good Clinical Practice Guidelines. Study participants provided written informed consent prior to study enrolment.

### p16 immunohistochemical staining

Deparaffinized sections were incubated with mouse anti-human p16 antibody (Clone E6H4) using the Ventana CINtext kit (Ventana Medical Systems, USA), according to the manufacturer’s instructions. Horseradish Peroxidase (HRP)-DAB staining was subsequently performed on the Ventana Benchmark XT Immunostainer (Ventana Medical Systems, USA) using the Ventana Optiview Amplification Kit (Ventana Medical Systems, USA).

### Preparation of blood plasma

Four 10 ml EDTA plasma tubes were collected and processed within one hour of collection. Tubes were centrifuged at 1,600 g for 20 minutes and further at 14,000 g for 10 minutes at room temperature to remove cellular debris and platelets. Samples are then stored in 1000 µL aliquots at −80 °C.

### Isolation and validation of EV

EV were isolated using two different methods to optimize the quality of extracted EV-DNA and EV-RNA yield recovery. EV-DNA was isolated using differential ultracentrifugation (UC), as this method demonstrated longer DNA fragment sizes (range: <100 to >10,000 bp) as per the Bioanalyzer High Sensitivity DNA kit analysis (Agilent Technologies, Australia). EV-RNA was isolated using ExoQuick ULTRA kit (System Biosciences, USA) as this method resulted in higher RNA yield (ranging between 15–25 pg/μl) as per the Bioanalyzer High Sensitivity RNA kit analysis (Agilent Technologies, Australia). For EV isolation using UC, 4000 µL clarified plasma was diluted in PBS and centrifuged at 12,000 g for 45 minutes, 110,000 g for 90 minutes, and finally at 110,000 g for 90 minutes at 4 °C using the Type 70 Ti rotor in the Optima™ L-90K Ultracentrifuge (Beckman Coulter, Australia). For EV isolation using the ExoQuick ULTRA kit (System Biosciences, USA, 2000µL clarified plasma was processed according to the manufacturer’s instructions. Validation of EV isolation in both methods was carried out according to guidelines recommended by the International Society of Extracellular Vesicles (ISEV) using Transmission Electron Microscopy (TEM), Nanoparticle Tracking Analysis (NTA), and Western blotting. For TEM, isolated EV were fixed, transferred onto 200 mesh Formvar-carbon coated copper grids (ProSciTech, Australia), and washed before being incubated with primary antibody (5 μl of mouse anti-human CD9, 10 μg/mL, Merck, Australia) and then with secondary antibody (5 μl of goat anti-mouse IgG-gold conjugate, 1:24, Aurion, The Netherlands). Grids were visualized on a JEM-2100 electron microscope (JEOL, Japan) and images were captured using an 11-megapixel Orius digital camera (Gatan, USA). For NTA, 100 μl of isolated EV were diluted in PBS and analyzed using a NS500 Nanoparticle tracking instrument (NanoSight NTA 3.0 Nanoparticle Tracking and Analysis Release Version Build 0064). Videos were captured using a 405 nm laser on a sCMOS camera. The instrument’s camera was set at level 15 and a threshold of three pixels. Settings for blur, minimum track length and minimum expected size were set to ‘auto’. A minimum of 200 completed tracks per video was collected for each analyzed sample. For Western blotting, 30 μl of isolated EV were mixed with RIPA (Sigma-Aldrich, USA) supplemented with a protease inhibitor cocktail (Roche Diagnostics, Australia). Extracted EV proteins were diluted in Lamelli Buffer (Bio-Rad, Australia), incubated and separated on a mini TGX 8–16% gel (Bio-Rad, Australia). Proteins were then transferred onto a nitrocellulose blotting membrane using the Trans-Blot® Turbo™ Transfer System (Bio-Rad, Australia). The membranes were blocked in 5% milk TBS before being probed with the following primary antibodies: TSG-101 (1:1000, clone EPR7130B, Abcam), CD9 (1:500, clone MM2/57, Life-Technologies), CD63 (1:1000, clone H5C6, BD-Biosciences). The membrane was subsequently probed with secondary antibodies (sheep anti-mouse IgG-HRP conjugate, polyclonal, 1:2000, GE Healthcare, donkey anti-rabbit IgG-HRP conjugate, polyclonal, 1:2000, GE Healthcare). Signals were detected with the GE healthcare Amersham™ ECL™ reagent and were subsequently imaged using the ChemiDoc™ Touch Imaging System (Bio-Rad, Australia).

### DNA and RNA extraction

EV-associated-DNA (EV-DNA) was extracted using the QIAamp DNA Microkit (Qiagen, Australia) according to the manufacturers’ instructions and EV-DNA was eluted in buffer AE. EV-associated-RNA (EV-RNA) was extracted from lysed vesicles eluted in QIAzol, and subsequently extracted using the RNA-extraction protocol of the ExoRNAeasy Serum/Plasma kit (Qiagen, Australia) according to the manufacturer’s instructions. EV-RNA was eluted in RNase-free water. From 2000 µL of plasma, cf-DNA and cf-RNA were extracted using the QIAamp Circulating Nucleic Acid Kit (Qiagen, Australia) according to the manufacturer’s instructions. Cf-DNA and cf-RNA were eluted in AVE buffer. Cf-RNA and EV-RNA were converted to cDNA using the SuperScript VILO cDNA Synthesis kit (Thermo Fisher Scientific, USA) according to the manufacturer’s instructions. DNA and RNA samples were stored at −80 °C until analysis.

### Circulating HPV-DNA and HPV-cDNA quantification using digital droplet polymerase chain reaction (ddPCR)

For cf-DNA and EV-DNA, amplifications were carried out in a 20 μL reaction containing 1× droplet digital PCR Supermix (no dUTP), 500 nM of HPV-subtype 16-E7 (HPV16E7) forward and reverse primers, 500 nM of HPV16-E7 probe, 900 nM of BRAF forward and reverse primers, 250 nM of BRAF probe as control, and 4.5 μL of cf-DNA or EV-DNA template. For cf-RNA and EV-RNA, amplifications were carried out in a 20 μL reaction containing 1× droplet digital PCR Supermix (no dUTP), 500 nM of HPV16-E7 forward and reverse primers, 500 nM of HPV16-E7 probe, 900 nM of BRAF*wtfl* forward and reverse primers, 250 nM of BRAF*wtfl* probe as control, and 4.5 μL of cf-cDNA or EV-cDNA template.

For HPV-DNA amplification, our protocol used HPV16-E7 primers, and probe sequences as per a previously described by Jeannot *et al*.^19^. The primers and probe for the HPV16-E7 sequence detection were 5′-TCCAGCTGGACAAGCAGAAC-3′ (forward primer), 5′CACAACCGAAGCGTAGAGTC-3’ (reverse primer), and 5′-FAM-ACAGAGCCCATTACAAT-3′ (Taqman probe). As gDNA control for the HPV-DNA assay, the primers and probe targeting BRAF intron-exon 15 were used as per the protocol described by Reid *et al*.^20^. The primers and probe for the BRAF sequence detection were 5′-CTACTGTTTTCCTTTACTTACTACTACACCTCAGA-3′ (forward primer), 5′-ATCCAGACAACTGTTCAAACTGATG-3′ (reverse primer), and 5′-VIC-CTAGCTACAGTGAAATC-MGBNFQ-3′ (Taqman). For HPV-RNA amplification, BRAF exon 4 and 5 spanning primers and probe were used as per a validated protocol designed within our laboratory. The primers and probe for the BRAF*wtfl* sequence detection were 5′-AATTGCATGTGGAAGTGTTG-3′ (forward primer), 5′-GCTTTCGACAAAAGTCACAA-3′ (reverse primer), and 5′-HEX-CACACAACT/ZEN/TTGTACGAA-3IABkFQ-3′ (Taqman probe). Droplets were generated and analyzed using the QX200 system (Bio-Rad, Australia). Amplifications were performed using the following conditions: one cycle of 95 °C for 10 minutes, 40 cycles of 94 °C for 30 seconds and 51 °C for one minute, and one cycle of 98 °C for 10 minutes. A positive control, negative control, healthy control and no template control were included in each run for cf-DNA/EV-DNA and cf-RNA/cf-RNA detection. Liver tissue was used as negative control, and serums collected from 10 healthy individuals with no prior HPV-driven pathologies were used as healthy controls. QuantaSoft version 1.6.6 analysis software (Bio-Rad, Australia) was used for data acquisition and analysis. The number of copies of HPV-DNA and HPV-RNA per 20 μl reaction was extrapolated to calculate copies per ml using volume of plasma (ml) used for DNA extraction, volume in which DNA was eluted (μl) and volume of DNA added to the PCR reaction (μl) as per calculation formulae published by Gray *et al*.^21^. EV-DNA samples were tested in triplicates due to the low concentration of EV-DNA available per reaction, whilst cf-DNA samples were tested in single runs due to its limited sample amount. An unevaluable reading is defined when there is less than 30 copies/ml of the reference BRAF DNA or BRAF*wtfl* cDNA present (i.e. less than three droplets detected). Detection sensitivity of circulating tumoral HPV-DNA/RNA in cf-DNA/RNA and EV-DNA/RNA was calculated as the number of patients with positively detected HPV-DNA, per number of known HPV-OPCSCC patients, excluding patients with unevaluable readings.

### Statistical analysis

To facilitate graphical representation and statistical analysis, samples with no detectable cf-DNA, EV-DNA, cf-RNA and EV-RNA were given a value of 1 copy/ml. Comparisons between cf-DNA/RNA and EV-DNA/RNA detection rates were performed using Fisher’s exact test, and comparisons between log-transformed values of circulating HPV-DN/RNA concentrations detected from different liquid biopsy sources were performed using unpaired t-test. Correlation between results was performed using the Pearson’s R test. Results were considered statistically significant at *p* < 0.05. Graphical and statistical analyses were conducted using Microsoft Excel®, version 16.16.11 and GraphPad PRISM ®, version 5.

### Translational relevance

Detection of circulating Human Papilloma Virus (HPV) in plasma presents a promising and non-invasive biopsy option for HPV-driven oropharyngeal squamous cell carcinoma (HPV-OPSCC) patients. Circulating extracellular vesicles (EV) containing tumoral DNA and RNA are a complimentary liquid biopsy source in addition to the established role of cell-free (cf)-DNA and cf-RNA. This study compared the detection sensitivity of HPV-DNA/RNA in EV-associated DNA/RNA and cf-DNA/RNA and showed that cf-DNA/RNA had a significantly higher rate of detection (91% vs. 42% in HPV-DNA, and 83% vs. 50% in HPV-RNA). Although our results showed that circulating HPV-DNA levels decreased in response to treatment in locally advanced disease following curative treatment, levels did not correlate to treatment response in metastatic disease. The study confirms the utility of cf-DNA/RNA as a source to assess the presence of tumoral HPV-DNA/RNA in plasma and its potential as a clinically useful biomarker.

## Results

### Patient characteristics

**Figure 1:**
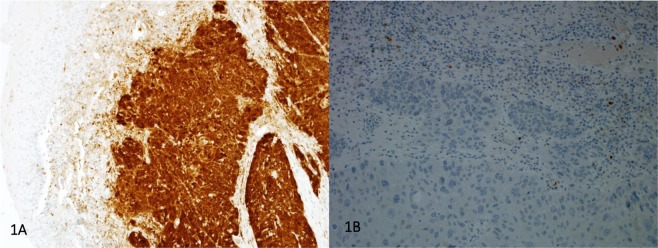
Examples of IHC staining of a p16 positive patient (1A) and of a p16 negative patient (1B).

A total of twenty-eight patients with base of tongue OPCSCC were included in this study (n = 28). Patient and tumour characteristics are outlined in Table 1. Twenty-two patients had locally advanced, and six patients with metastatic disease. Eighteen out of 22 locally advanced (stage I-IVB, 7^th^ AJCC staging criteria), and 5 out of 6 metastatic (stage IVC, 7^th^ AJCC staging criteria) patients were p16 IHC positive (Fig. 1A), giving a total of 23 HPV-OPCSCC cases, whilst four locally advanced and one metastatic patients had p16 IHC negative disease (Fig. 1B). The median age was 63 years old (range: 46–78). The majority of patients were male (26/28, 93%), and of Caucasian descent (28/28, 100%). In the locally advanced patient cohort, all patients had curative intent treatment with the majority receiving radical chemoradiation (20/22, 91%) for stage IVA disease (18/22, 82%), and were current or ex-smokers (14/22, 64%). In the metastatic patient cohort, patients received palliative radiation, chemotherapy or immunotherapy. The majority in this cohort were current or ex-smokers (4/6, 67%). The median follow up duration from the date of patient consent to the time of data cut-off was 20 months (range: 1–43 months).

**Table 1:** Patient and tumour characteristics.

Characteristics	Total (*n* = 28)	Locally advanced (*n* = 22)	Metastatic (*n* = 6)
**p16 IHC status**			
Positive	23 (82%)	18 (82%)	5 (83%)
Negative	5 (18%)	4 (18%)	1 (17%)
**Age**			
Median (range) years	63 (46–78)	64 (46–78)	62 (54–64)
**Gender**			
Female	2 (7%)	0 (0%)	2 (33%)
Male	26 (93%)	22 (100%)	4 (67%)
**Race**			
Caucasian	28 (100%)	28 (100%)	28 (100%)
Other	0 (0%)	0 (0%)	0 (0%)
**Stage (7**^**th**^ **AJCC edition)**			
III	2 (7%)	2 (9%)	0 (0%)
IVA	18 (64%)	18 (82%)	0 (0%)
IVB	2 (7%)	2 (9%)	0 (0%)
IVC	6 (22%)	0 (0%)	6 (100%)
**Smoking or tobacco use**			
Ex-smoker	11 (39%)	8 (36.5%)	3 (50%)
Current smoker	7 (25%)	6 (27%)	1 (17%)
Non-smoker	10 (36%)	8 (36.5%)	2 (33%)

### Validation of isolated EV

**Figure 2:**
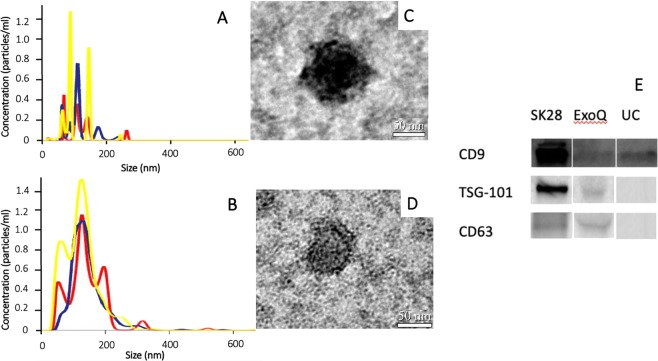
Validation and characterization of isolated EV: Size and concentration distribution of nanoparticles from NTA in triplicate (yellow, red and blue graphs) (1 A – UC; 1B – ExoQuick); Immunogold labelled TEM images (1C – UC; 1D – ExoQuick); and Western blots cropped from different parts of the same gel (lane 2 – ExoQuick; lane 3 – UC) using SKMel28 Cell Lysate as positive control (lane 1) (1E).

Validation of EV isolation from UC and ExoQuick was carried out using Western blot, TEM and NTA, with collective results presented in Fig. 2. NTA results in Fig. 2A (UC) and Fig. 2B (ExoQuick) of isolated EV analyzed in triplicate (yellow, red and blue graphs) had a mean particle size of 113.3 nm and 134.9 nm, and mean concentration of 4.2×10^10^ particles/ml and 1.8×10^9^ particles/ml respectively. Figure 2C (UC) and Fig. 2D (ExoQuick) are immunogold labelled TEM images of two representative isolated EV at magnification x25000, which showed round particles with diameters of approximately 100 nm and 60 nm respectively. Western blot results, using melanoma cell line lysate (SKMel28) as positive control (Fig. 2E – as groupings of blots cropped from different parts of the same gel) show UC-isolated EV populations were positive for CD9, a common EV marker, and ExoQuick-isolated EV populations were positive for CD9, and also TSG-101, and CD63, two other common EV markers. Full length gel is presented in Supplementary Data.

### Circulating HPV-DNA detection in cf-DNA and EV-DNA

**Figure 3:**
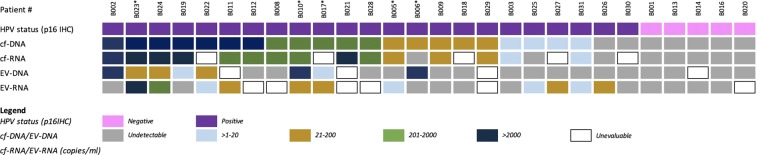
Summary of patient characteristics and circulating HPV-DNA and HPV-RNA of HPV-subtype 16 quantification in cf-DNA/RNA and EV-DNA/RNA, relative to HPV status in the tumor based on p16 IHC positivity. *Indicates patients with metastatic disease.

**Figure 4:**
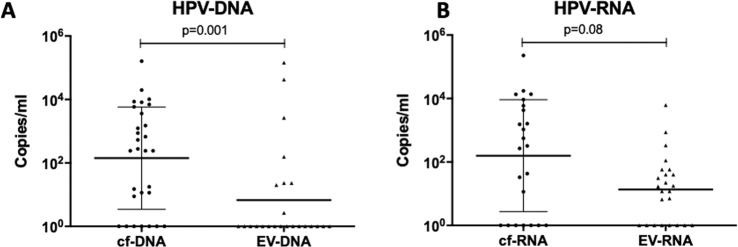
**(A,B)** Circulating HPV-DNA and HPV-RNA levels (copies/ml) detected in cf-DNA/RNA and EV-DNA/RNA fractions, respectively.

Figure 3 summarizes the circulating HPV-DNA and HPV-RNA of HPV-subtype 16 quantification results relative to p16 IHC status, followed by detected copies/ml levels in cf-DNA, cf-RNA, EV-DNA, and EV-RNA. All cf-DNA samples were evaluable, containing between 660–113,860 copies/ml of reference BRAF DNA as the equivalent of genome copies. We were able to detect baseline circulating HPV-DNA in cf-DNA from 21 out of 23 HPV-OPCSCC cases, resulting in a detection sensitivity of 91%. In comparison, we only detected circulating HPV-DNA in EV-DNA from eight out of 19 HPV-OPCSCC evaluable cases (with four samples deemed unevaluable), containing between 11–39,689 copies/ml of reference BRAF DNA as the equivalent of genome copies, resulting in a detection sensitivity of 42%. Thus, circulating tumoral HPV-DNA detection rate was statistically significantly higher in cf-DNA compared to EV-DNA (p < 0.001). There were no cases of positive circulating HPV-DNA detection with either cf-DNA or EV-DNA in patients with negative p16 IHC status (n = 5). Albeit the small number of samples, this suggests a 100% specificity. HPV-DNA levels were statistically significantly higher in cf-DNA (median: 880 copies/ml, range: 11–161,680 copies/ml) than in EV-DNA (median: 156 copies/ml, range: 2–144,660 copies/ml) (*p* = 0.001) (Fig. 4A). There was poor correlation between baseline circulating HPV-DNA levels in cf-DNA and EV-DNA (*r* = −0.057).

### HPV-RNA detection in cf-RNA and EV-RNA

Twenty-three patients’ samples of cf-RNA and EV-RNA were evaluable, containing between 320–28,220 copies/ml and 10–316 copies/ml of reference BRAF*wtfl* cDNA as the equivalent of genome copies, with five samples having too low reference *BRAFwtfl* cDNA to be deemed evaluable. Circulating HPV-RNA transcripts of HPV-subtype 16 were detected in cf-RNA from 15 out of 18 HPV-OPCSCC evaluable cases, with a detection sensitivity of 83%. In comparison, HPV-RNA transcripts of HPV-subtype 16 were detected in EV-RNA in 9 out of 18 HPV-OPCSCC evaluable cases, resulting in a detection sensitivity of 50%. Thus, the detection of circulating tumoral HPV-RNA of was also significantly higher in cf-RNA compared to EV-RNA (*p* = 0.0019). No circulating HPV-RNA was detected in patients with a negative p16 IHC status (n = 5) suggesting a 100% specificity. HPV-RNA levels were not significantly different between cf-RNA (median: 120 copies/ml, range:12–228,440 copies/ml) and EV-RNA (median: 12 copies/ml, range:7–6220 copies/ml, *p* = 0.08) (Fig. 4B). All cases that had HPV-RNA in cf-RNA detected also had HPV-DNA detected in cf-DNA (Fig. 3). However, there were three cases (patients #B025, #B026, #B031) that had detectable HPV-RNA in EV-RNA that did not have HPV-DNA detectable in EV-DNA (Fig. 3). One of the three cases (patient ID #B026) also had undetectable HPV-DNA in cf-DNA, and undetectable HPV-RNA in cf-RNA. There was a strong correlation between baseline circulating HPV-RNA levels in cf-RNA and EV-RNA values (*r* = 0.98, *p* < 0.001).

### Circulating HPV-DNA levels in cf-DNA and EV-DNA decrease in locally advanced HPV-OPCSCC patients after completion of curative therapy

**Figure 5:**
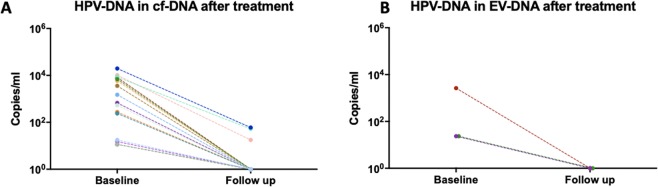
**(A,B)** Circulating HPV-DNA levels (copies/ml) in 16 evaluable cf-DNA samples (4A) and three evaluable EV-DNA samples (4B) of patients with locally advanced HPV-OPCSCC disease at baseline (pre- curative treatment) and at follow-up (post- curative treatment).

In the locally advanced HPV-OPCSCC cohort, 16 out of 18 patients had detectable circulating HPV-DNA in cf-DNA at baseline. Follow up HPV-DNA analysis in these 16 patients at 6–12 weeks post completion of curative treatment revealed a reduction in HPV-DNA levels in all cases (100%), with 13 (81%) patients having undetectable HPV-DNA levels in plasma (Fig. 5A). Three patients (19%) had residual detectable levels post-treatment but remain disease-free at a median follow up of 20 months. When HPV-DNA in EV-DNA was evaluated, only six patients out of 18 patients in the cohort had detectable levels at baseline, and only three patients had evaluable longitudinal EV-associated-DNA samples. Of these, all had undetectable transcripts level at six weeks and beyond post- completion of curative treatment (Fig. 5B). Two out of these three patients also had undetectable HPV-DNA levels using cf-DNA, however one patient with undetectable HPV-DNA level using EV-DNA, had residual detectable HPV-DNA levels using cf-DNA.

### Changes in circulating HPV-DNA levels in cf-DNA and EV-DNA in metastatic HPV-OPSCSS patients during therapy

**Figure 6:**
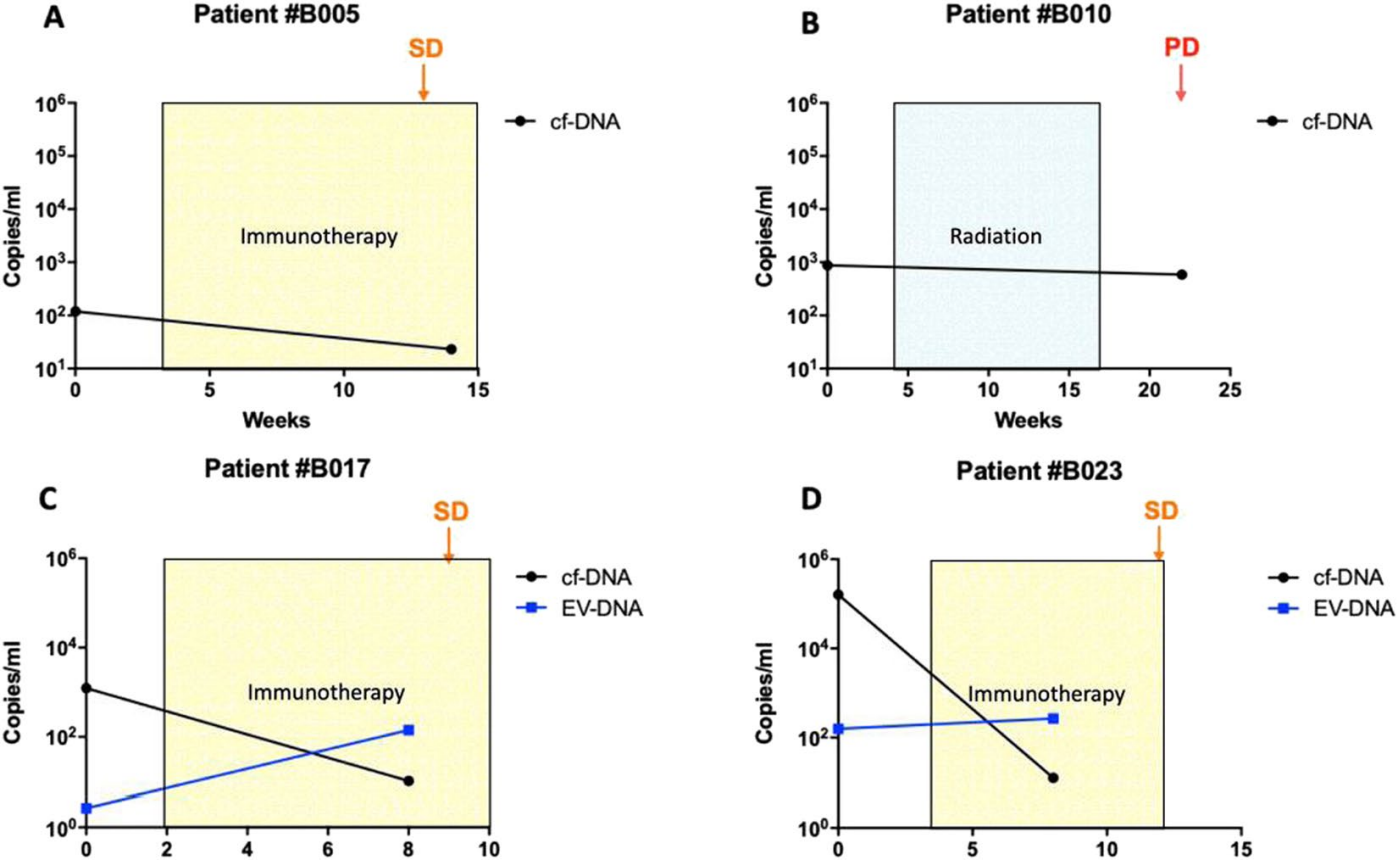
Changes in circulating HPV-DNA levels (copies/ml) in four metastatic patients during treatment. Disease was assessed using whole-body CT scans with SD denoting stable radiological disease and PD denoting radiological progressive disease according to RECIST 1.1 criteria with specific tumour response evaluation measurements as below. (**A**) Patient #B005: SD was defined by 0% change to sum of diameters of target lesions according to RECIST 1.1. The patient received programmed cell death-1 (PD-1) inhibitor immunotherapy. (**B**) Patient #B010: PD was defined by the appearance of a new target lesion according to RECIST 1.1. Patient received treatment of radiation to the primary base of tongue cancer. **C**) Patient #B017: SD was defined as a 9% increase in the sum of diameter of target lesions according to RECIST 1.1. The patient received PD-1 inhibitor based immunotherapy. (**D**) Patient #B023: SD was defined by a 17% increase in the sum of diameters of the target lesions according to RECIST 1.1. The patient received PD-L1 inhibitor immunotherapy.

Out of six patients in the metastatic HPV-OPCSCC cohort, longitudinal data of HPV-DNA levels were evaluable in four cf-DNA patients and in two EV-DNA patients. Changes in HPV-DNA levels, treatments, and disease progress assessed by RECIST 1.1 criteria with specific tumour response evaluation measurements are detailed in Fig. 6. Circulating HPV-DNA levels did not consistently increase or decrease according to treatment response. For example, circulating HPV-DNA levels in cf-DNA decreased at the time of stable disease (SD) in three patients (patients #B005, #B017 and #B023) but also decreased despite disease progression (PD) in patient #B010. Similarly, circulating HPV-DNA levels of EV-DNA increased in a patient found to have SD (patient #B017). There was no concordance in findings between cf-DNA and EV-DNA longitudinal changes during treatment.

## Discussion

There is an increasing clinical need to improve the detection technology in OPCSCC, and liquid biopsy technologies such as cf-DNA and EV-DNA present novel non-invasive means to detect circulating HPV viral element in patients’ biofluids. To the best of our knowledge, this study is the first to report on the detection of circulating HPV in EV-DNA/RNA in patients with HPV-OPCSCC. Overall, our study found that cf-DNA/RNA had statistically higher detection sensitivity for circulating tumoral HPV-DNA and HPV-RNA compared to EV-DNA/RNA (*p* = < 0.001 and *p* = 0.0019, respectively).

The use of cf-DNA for the detection of circulating HPV-DNA has been investigated in a number of other studies and cancer types, with reported detection sensitivities that are similar to our findings (91%). In different HPV-induced malignancies, detection sensitivities of cf-DNA analyses have been reported to range from 86%-100% in anal SCC (n = 19 and n = 57), cervical SCC (n = 33), and head and neck SCC (n = 93, including oral cavity, oropharynx, hypopharynx and larynx subsites)^22–25^. For HPV-OPCSCC specifically, three studies have demonstrated detection sensitivities of 95.6% (n = 97), 89% (n = 103), and 88.5% (n = 114)^26–28^. Albeit the small cohort of patients, our study with a small cohort of patients showed that none of the HPV-negative OPCSCC patients had circulating HPV-DNA in plasma, representing a specificity of 100%. Our study contributes to the growing body of data that indicates that that plasma-derived cf-DNA is a reliable source for circulating HPV-DNA detection. We also showed that cf-RNA is another source of tumoral HPV-RNA assessment, with a slightly lower detection sensitivity level of 83%. This is of particular interest, as RNA detection reflects the transcriptionally active viral element. Again, to the best of our knowledge, our study is the first to investigate detection sensitivity of circulating HPV-RNA in liquid biopsy sources.

EV assessment has been of interest given that EV are hypothesized to contain more intact molecular contents actively secreted by dividing cells compared to the more fragmented, apoptotic cellular materials derived from cell-free circulating nucleic acids^12–16^. Furthermore, although EV has been implicated in both HPV-transmission and carcinogenesis, they have not been demonstrated to carry HPV-molecular elements outside of the pre-clinical settings, and thus our study presents novel findings that circulating HPV-DNA/RNA can be detected in EV-DNA/RNA^29^. However, overall our data demonstrates that cf-DNA assessment is more sensitive compared to EV-DNA assessment for the detection of circulating HPV-DNA. This is important given that cfDNA assessment is well established with optimized extraction methodology and validated, reproducible streamlined protocols already in routine clinical use. In contrast, our study demonstrated discrepancies in size, concentration, and sub-population between EV isolated from two different methods UC and ExoQuick. Different EV-isolation methods produced different yields and qualities of EV-DNA and EV-RNA, and further challenge the lack of existing consensus regarding the optimal extraction methodology for EV isolation. The more commonly used method of UC can be laborious and not consistently reproducible^30,31^.

In the context of HPV-DNA clearance in locally advanced patients undergoing curative intent treatments, our study demonstrated that circulating HPV-DNA levels in cf-DNA and EV-DNA decreased in 100% of patients, with 81% of patients’ HPV-DNA levels dropping to undetectable levels in cf-DNA. With a median follow up of 20 months, all patients are disease-free, including the three patients with residual detectable cf-DNA. The correlation between the treatment response and dynamic changes in cf-DNA levels are consistent with two previously published HPV-OPCSCC studies (n = 97 with follow-up duration not reported and n = 103 with median follow-up time of 16.5 months)^26,27^.

In the context of metastatic disease, published longitudinal data is limited. Circulating HPV-DNA levels have been reported in three small studies in HPV-driven cancers to have changes consistent with the disease burden and treatment response^22,32,33^. This is in contrast with the results found in our study, which did not find any correlation between circulating HPV-DNA levels correlating with disease response to treatment. The three published studies used ddPCR to detect circulating HPV-DNA, and included patients with metastatic HPV-driven cervical cancer (n = 19); a case report in metastatic HPV-driven anal cancer (n = 1); and metastatic HPV-OPCSCC (n = 22)^22,32,33^. It is worth noting that these studies all used different definitions of tumor burden measurements to our study; one used RECIST 1.0 criteria, one used immune-related total measurable tumor burden, and one used another novel measurement method to derive the total tumour burden. We utilized the well-recognized and more recent RECIST 1.1 criteria. The difference in defining tumour burden measurements may explain in part the contrasting results of the three published studies and ours. Although our findings in the metastatic cohort should be interpreted cautiously given the limited sample size (n = 4), the lack of correlation with treatment response in even these few patients heralds the need for future research, particularly in patients receiving immunotherapy. Similar to other blood-based biomarkers, circulating HPV-DNA levels may not consistently correlate with disease burden or treatment response for all patients.

Our study is limited by the small cohort size. The accuracy of HPV-DNA/RNA detection rate in both cf- and EV DNA/RNA would be improved if investigated in larger cohorts. Additionally, longitudinal circulating HPV-DNA data in the metastatic cohort was also limited to between 2–5.5 months in four patients and was only assessed over two time points. More data points and with a longer follow-up will allow a more in-depth analysis of whether these changes continue to remain inconsistent with disease response or may represent early molecular disease progression prior to detection on conventional imaging. The isolation of EV-RNA from plasma was challenging, evidenced by low levels of reference BRAF*wtfl* cDNA consistent with poor yield, which may have underestimated its detection sensitivity. This is an ongoing challenge for liquid biopsy approaches, with previously published data of EV-RNA concentrations being generally lower than DNA yields, resulted by the fact that RNA is highly susceptible to degradation^34^.

## Conclusion

Our findings confirm the clinical utility of cf-DNA/RNA and EV-DNA/RNA as valid tests for the detection of circulating tumoral HPV-DNA/RNA. Given the significantly higher detection rate and practicality, our study suggests that cf-DNA assessment is superior to EV-DNA assessment. Additional studies are required in other cancer types and larger cohorts comparing cf-DNA and EV-DNA analyses to clarify whether cf-DNA assessment as a liquid biopsy source is consistently and universally superior. Our study confirms clearance of tumoral HPV-DNA in patients with locally advanced disease following curative treatment. However, HPV-DNA level dynamics in patients with metastatic disease during palliative treatment did not appear clinically useful. Further studies are required in larger HPV-OPSCC cohorts with appropriate follow up to define the patients and clinical setting for which assessment of circulating tumoral HPV-DNA levels in cf-DNA will be a clinically useful disease monitoring biomarker.

## Supplementary information

Supplementary Information.
