# Supplementary material for: A comparative study of extracellular vesicle-associated and cell-free DNA and RNA for HPV detection in oropharyngeal squamous cell carcinoma
Source: Sci Rep. 2020 Apr 8;10:6083. doi: 10.1038/s41598-020-63180-8 (PMC7142128; doi:10.1038/s41598-020-63180-8)
Supplement: Supplementary file 1 — Supplementary Information. [file 41598_2020_63180_MOESM1_ESM.pdf]

Supplementary data

Study title: A comparative study of extracellular vesicle-associated and cell-free DNA and RNA for HPV detection in oropharyngeal squamous cell carcinoma.

Authors:

Bella Nguyen[1,2], Katie Meehan[2,3], Michelle R Pereira[4], Bob Mirzai[2,7], Si Hong Lim[5], Connull Leslie[6,7], Michael Clark[4], Chady Sader[8,9], Peter Friedland[8,10,11], Andrew Lindsay[12], Colin Tang[4,13], Michael Millward[1,14], Elin Gray[4], Annette M Lim[1,2,15]

Affiliations:

1. *Department of Medical Oncology, Sir Charles Gairdner Hospital, Perth, Western Australia, Australia*
2. *School of Biomedical Sciences, University of Western Australia, Perth, Western Australia, Australia*
3. *Chinese University of Hong Kong, Shatin, Hong Kong.*
4. *School of Medical and Health Sciences, Edith Cowan University, Joondalup, Western Australia, Australia*
5. *Genomics Western Australia, Telethon Kids Institute, Western Australia, Australia.*
6. *School of Pathology and Laboratory Medicine, The University of Western Australia, Perth, Western Australia, Australia*
7. *Department of Anatomical Pathology, PathWest, QEII Medical Centre, Perth, Western Australia, Australia*
8. *Department of Otolaryngology, Head and Neck Surgery, Sir Charles Gairdner Hospital, Perth, Western Australia, Australia*
9. *Department of Otolaryngology, Head and Neck Surgery, St John of God Murdoch Hospital, Perth, Western Australia, Australia*
10. *Faculty of Medical and Health Sciences, University of Western Australia, Perth, Western Australia, Australia*
11. *School of Medicine, University of Notre Dame, Fremantle, Western Australia, Australia.*
12. *Department of Otolaryngology, Head and Neck Surgery, Hollywood Private Hospital, Perth, Western Australia, Australia*
13. *Department of Radiation Oncology, Sir Charles Gairdner Hospital, Perth, Western Australia, Australia*
14. *School of Medicine, The University of Western Australia, Perth, Western Australia, Australia*
15. *Institute for Health Research, University of Notre Dame, Fremantle, Western Australia, Australia.*

Corresponding author:

Dr Annette M Lim
Email: Annette.Lim@health.wa.gov.au
Address: Department of Medical Oncology, Sir Charles Gairdner Hospital, B Block, Hospital Ave, NEDLANDS, WA, 6009.
Telephone: (+61) 8 6457 3333; Facsimile: (+61) 8 6457 1018

Western blot Original Gel

TSG-101

CD81

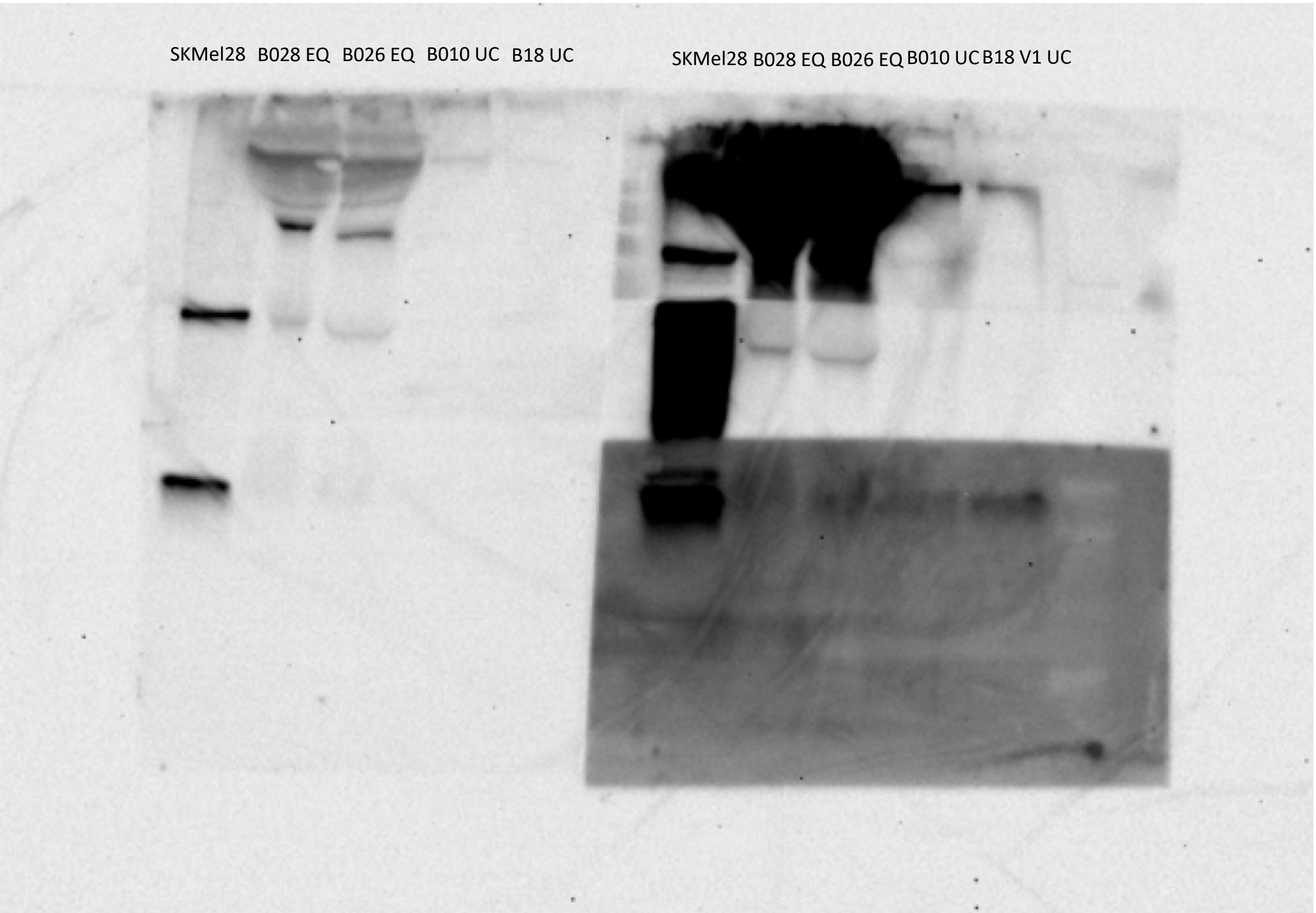

Calnexin

CD63

CD9

Legend:
SKMel28 Cell lysate as positive control
EQ = ExoQuick sample
UC = Ultracentrifugation sample

ChemiDoc™ Touch Imaging System (Bio-Rad, Australia)

Western blot gel: Exposure used for CD9, TSG101 and CD81

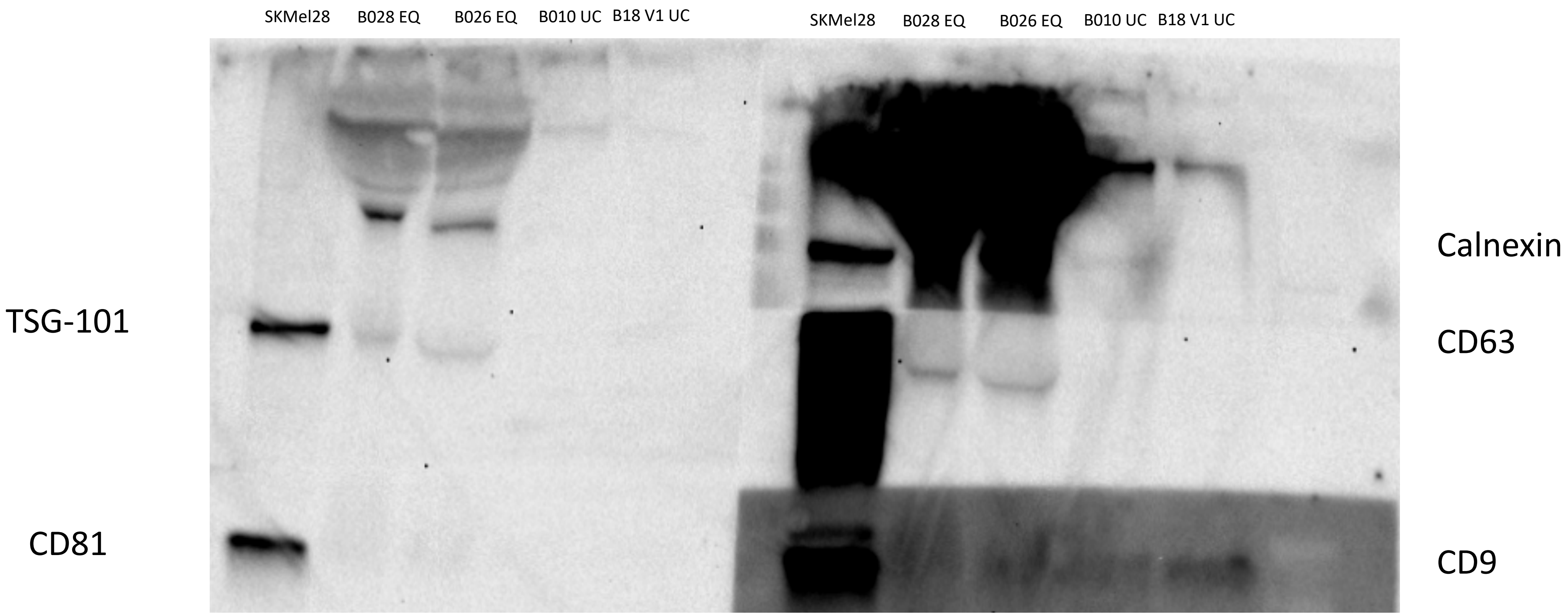

Legend:
SKMel28 Cell lysate as positive control
EQ = ExoQuick sample
UC = Ultracentrifugation sample

ChemiDoc™ Touch Imaging System (Bio-Rad, Australia)

Western blot: Exposure used for CD63 and Calnexin (180S)

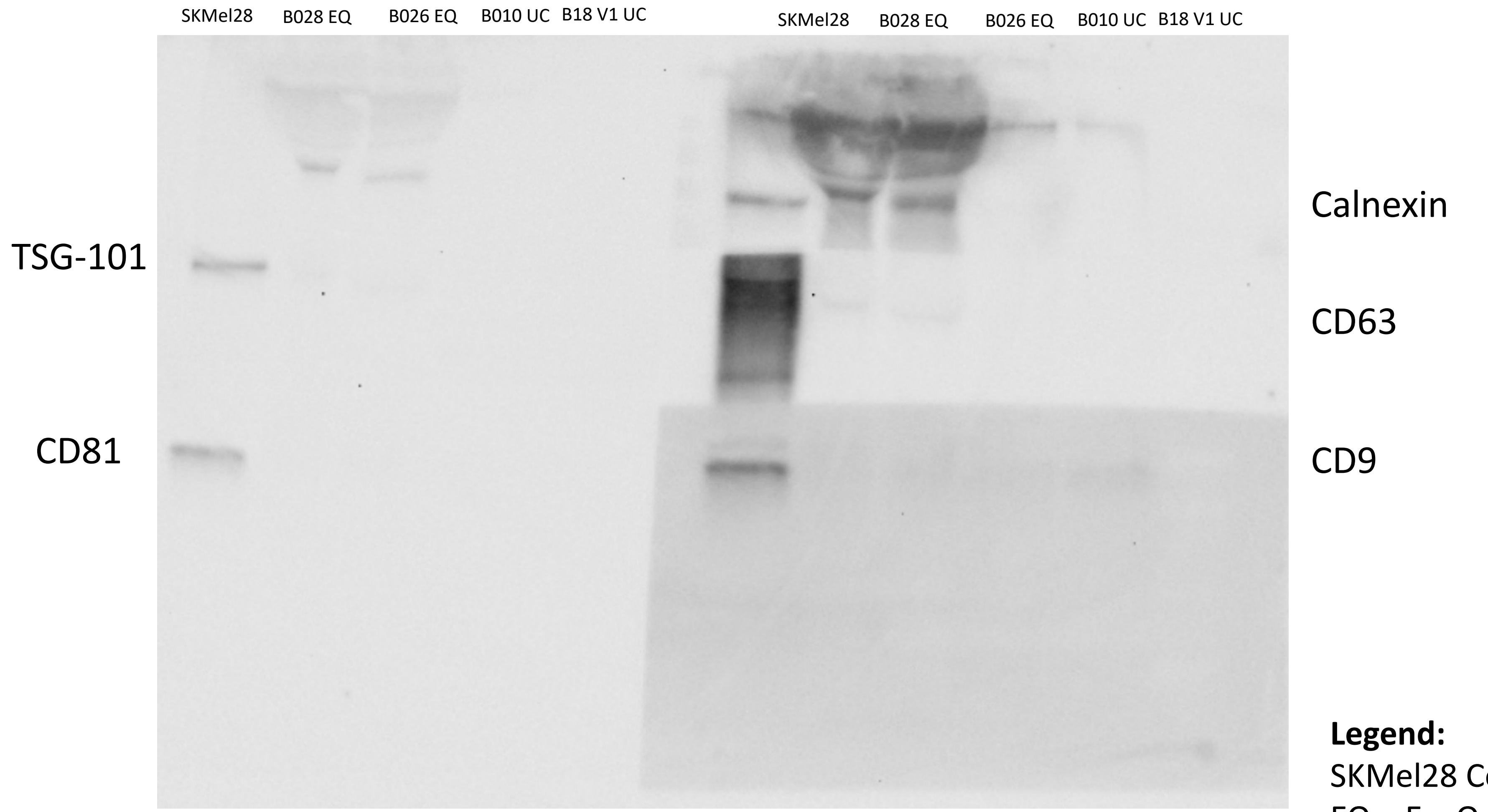

Legend:
SKMel28 Cell lysate as positive control
EQ = ExoQuick sample
UC = Ultracentrifugation sample

ChemiDoc™ Touch Imaging System (Bio-Rad, Australia)

Western blot molecular weight ladders

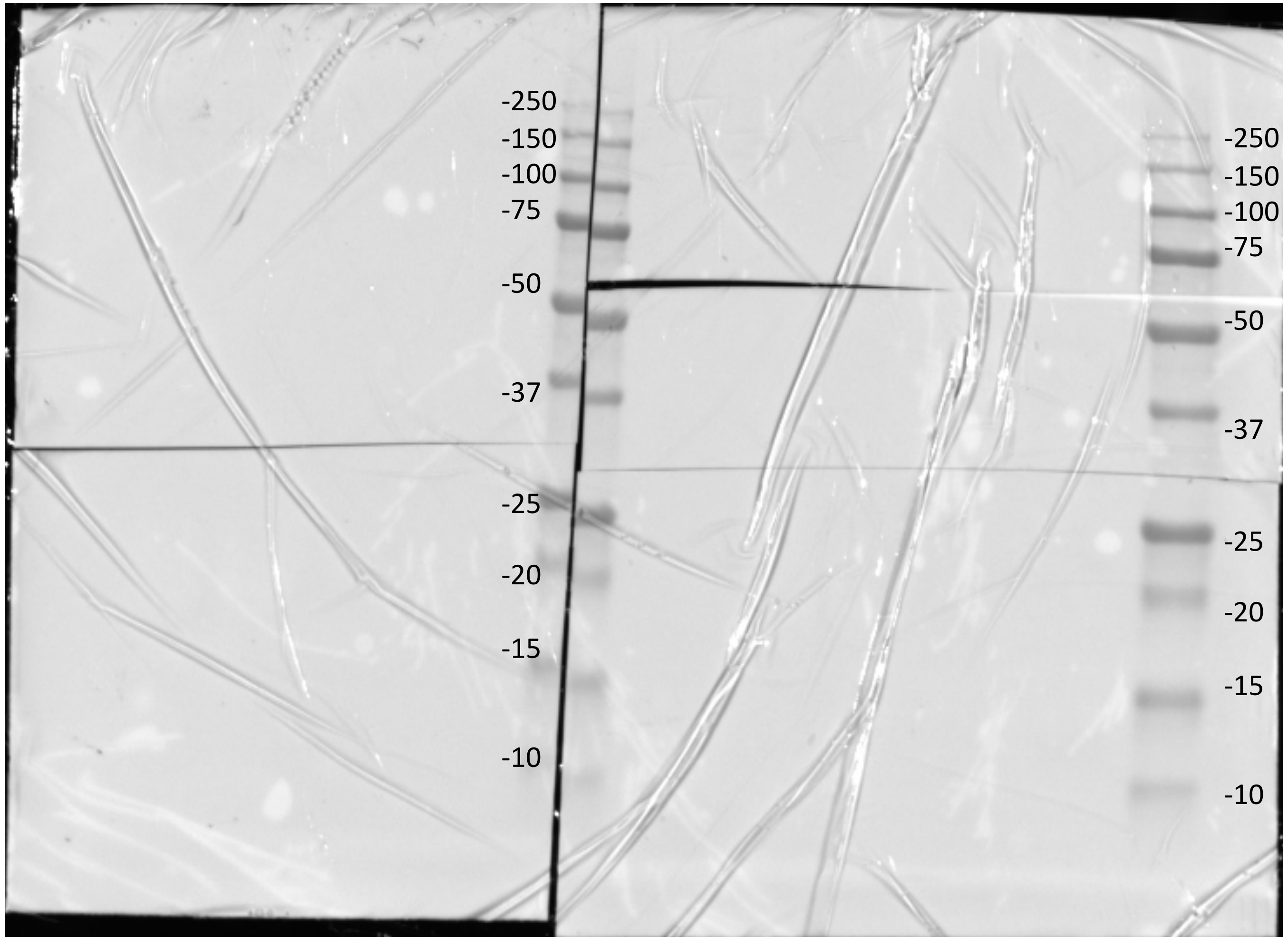

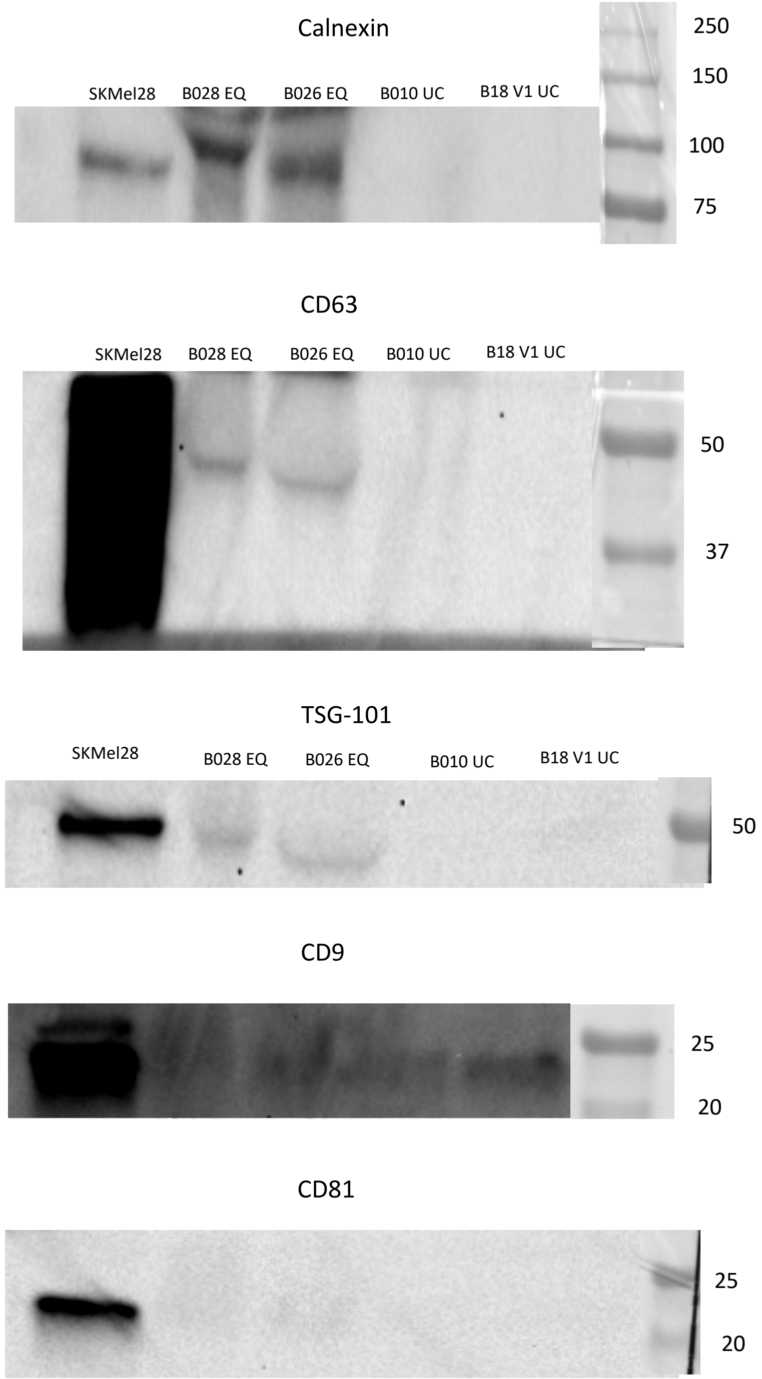

Summary of cropped results with ladder
